# Multivariate Analysis and Validation of the Prognostic Factors for Skull Base Chordoma

**DOI:** 10.3389/fsurg.2021.764329

**Published:** 2021-11-23

**Authors:** Chubei Teng, Qi Yang, Zujian Xiong, Ningrong Ye, Xuejun Li

**Affiliations:** ^1^Department of Neurosurgery, Xiangya Hospital, Central South University, Changsha, China; ^2^Hunan International Scientific and Technological Cooperation Base of Brain Tumor Research, Xiangya Hospital, Central South University, Changsha, China; ^3^Department of Neurosurgery, The First Affiliated Hospital, University of South China, Hengyang, China

**Keywords:** gross total resection, nomogram, SEER program, skull base chordoma, subtotal resection

## Abstract

**Background:** Skull base chordoma is a rare tumor with low-grade malignancy and a high recurrence rate, the factors affecting the prognosis of patients need to be further studied. For that, we investigated prognostic factors of skull base chordoma through the database of the Surveillance, Epidemiology, and End Results (SEER) program, and validated in an independent data set from the Xiangya Hospital.

**Methods:** Six hundred and forty-three patients diagnosed with skull base chordoma were obtained from the SEER database (606 patients) and the Xiangya Hospital (37 patients). Categorical variables were selected by Chi-square test with a statistical difference. Survival curves were constructed by Kaplan–Meier analysis and compared by log-rank test. Univariate and multivariate Cox regression analyses were used to explore the prognostic factors. Propensity score matching (PSM) analysis was undertaken to reduce the substantial bias between gross total resection (GTR) and subtotal resection (STR) groups. Furthermore, clinical data of 37 patients from the Xiangya Hospital were used as validation cohorts to check the survival impacts of the extent of resection and adjuvant radiotherapy on prognosis.

**Results:** We found that age at diagnosis, primary site, disease stage, surgical treatment, and tumor size was significantly associated with the prognosis of skull base chordoma. PSM analysis revealed that there was no significant difference in the OS between GTR and STR (*p* = 0.157). Independent data set from the Xiangya Hospital proved no statistical difference in OS between GTR and STR groups (*p* = 0.16), but the GTR group was superior to the STR group for progression-free survival (PFS) (*p* = 0.048). Postoperative radiotherapy does not improve OS (*p* = 0.28), but it can prolong PFS (*p* = 0.0037). Nomograms predicting 5- and 10-year OS and DSS were constructed based on statistically significant factors identified by multivariate Cox analysis. Age, primary site, tumor size, surgical treatment, and disease stage were included as prognostic predictors in the nomograms with good performance.

**Conclusions:** We identified age, tumor size, surgery, primary site, and tumor stage as main factors affecting the prognosis of the skull base chordoma. Resection of the tumor as much as possible while ensuring safety, combined with postoperative radiotherapy may be the optimum treatment for skull base chordoma.

## Introduction

Chordoma, derived from embryonic notochord remnant tissue, is a low-grade malignant tumor, with an incidence of about 0.08/100,000 (1). It accounts for 1–4% of all primary bone tumors and mainly occurs in the axial bone of the skull base region (32%), the sacrococcygeal region (29.2%), the spine (32.8%), and the other 6.0% chordoma were found outside the axial bone (2). As its special location and relatively poor prognosis, skull base chordoma which usually occurs in the spheno-occipital region is the research hotspot among chordoma.

The pathology of skull base chordoma does not show many characteristics of malignancy tumors, and the tumor proliferation rate is not high, but local invasiveness is strong and the recurrence rate is high (3). Due to the skull base chordoma's slow progressive growth and lack of specific symptoms in the early stage, the tumor is often quite large when symptoms appear. Surgical resection is the primary choice for the treatment of skull base chordoma; however, its deep site which is adjacent to the brain stem, important cranial nerves, and intracranial vessels makes it difficult for surgeons to remove the tumor safely and completely. Radiotherapy is an important adjuvant treatment, chordoma is insensitive for low-dose radiotherapy, it needs high-dose radiotherapy to kill tumor cells, but high doses of radiation may damage surrounding brain tissue and nerves, limiting the use of radiotherapy in chordoma treatment (4). However, the median survival of patients who have received treatments was 6.29-years, the 5- and 10-year survival rates were 67.6 and 39.9%, respectively (5). Therefore, it is necessary to explore the prognostic factors for skull base chordoma.

Previous studies have shown that factors affecting prognosis include patient's age, tumor size, tumor site, and vascular involvement (5–7). Meanwhile, some molecular markers such as high expression of Ki-67 and MIB-1 are associated with poor prognosis and high recurrence rate (8–10). However, the effect of the extent of surgical resection on skull base chordoma prognosis is not clear and needs to be further studied. On the one hand, aggressive total resection may lead to serious complications that may reduce the survival rate, and different centers reported different total resection rates, the average total resection rate being only 24–52% (11–13). On the other hand, incomplete resection will increase the recurrence rate (14). Therefore, whether gross total resection (GTR) is necessary and whether subtotal resection (STR) will affect the survival rate remains to be further studied.

**Figure 1:**
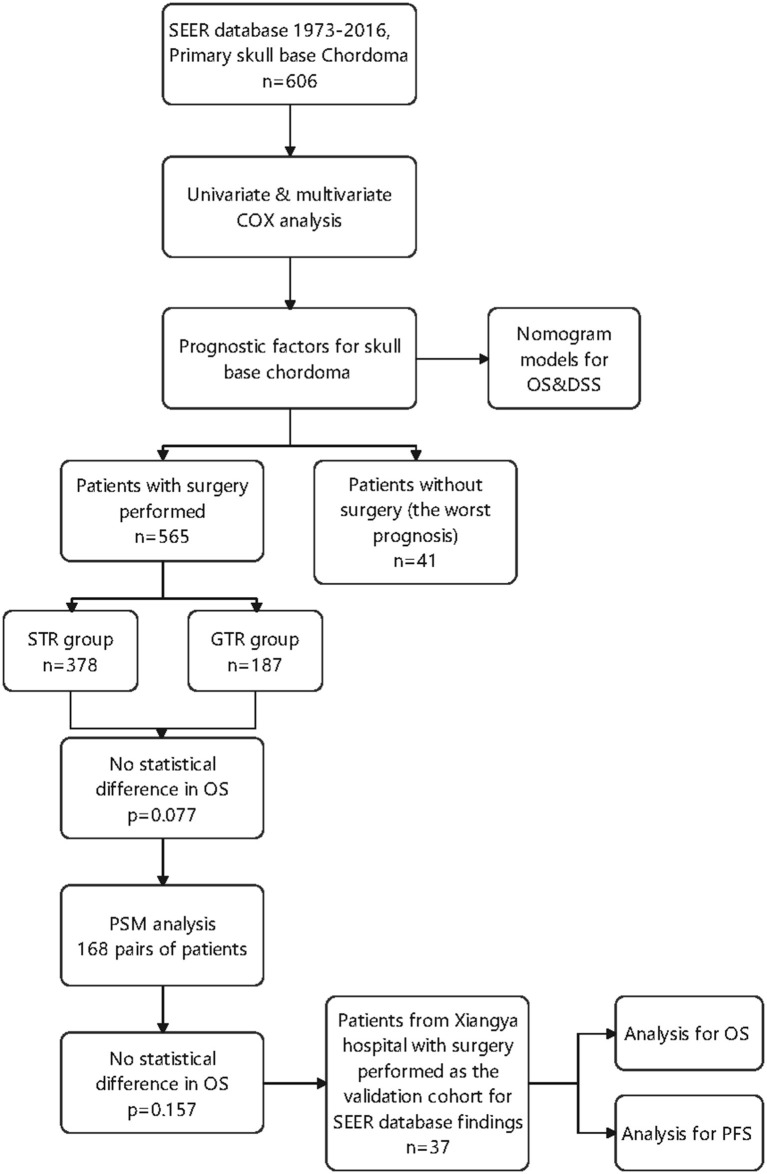
The flow chart of data collection and analysis procedure in this study.

In this study, we analyzed prognostic factors in 606 patients with skull base chordoma based on clinical data from the Surveillance Epidemiology and End Results (SEER) database sponsored by the American Cancer Institute, with a focus on evaluating the effect of surgical resection extent and radiotherapy on patients' prognosis. After that, we brought in clinical data from our center to validate our findings. Furthermore, we attempted to establish prediction models using the line diagram. A flow chart was drawn to show the data collection and analysis procedure in this study (Figure 1). By this study, we hope to provide treatment strategies for clinicians and serve as a basis for further research on skull base chordoma.

## Materials and Methods

### Data Collection and Processing

Patients with a diagnosis of skull base chordoma between 1973 and 2016 were selected from the SEER database (SEER^*^Stat software version 8.3.6). The International Classification of Diseases for Oncology Third Edition (ICD-O-3) was used to identify histologically confirmed cases of chordoma, chondroid chordoma, and dedifferentiated chordoma with the codes 9370, 9371, and 9372. Chordomas in the bones of skull and face (C41.0), brain (C71.0—71.9), pituitary gland (C75.1), or other head structures (C10.0—C10.9, C11.0—C11.9, C14.0, C30.0, C31.3, C31.9, C49.0, C70.0, C72.5, C76.0), were taken to represent skull base lesions. Information of patients, including age at diagnosis, race, gender, year of diagnosis, marital status, primary tumor sites, tumor size, disease stage, radiation record, chemotherapy record, follow-up information, and extent of surgical resection, was collected in this study. According to the information on the extent of surgical resection obtained from the SEER database, we characterized “total resection,” “radical resection,” and “gross resection” as GTR, and “local excision,” “partial excision,” and “subtotal resection” as STR. Patients who were coded as “not primary or first tumor” and “surgery not otherwise specified” were excluded from the survival analysis.

To validate the results of the SEER cohort, 37 patients who have been diagnosed with skull base chordoma and received surgery at the Xiangya Hospital between 2010 and 2020 were selected for retrospective analysis. The study was approved by the Ethics Committee of Xiangya Hospital of Central South University (IRB NO. 202012229). Written informed consent for patients included in the study was obtained. The extent of surgical resection was determined based on postoperative imaging data and surgical records. GTR was defined as total resection of the tumor with no image contrast-enhanced, STR was defined as incomplete resection of the tumor with more than 90% resection (15). Patients were followed up until February 2021. Overall survival (OS) is defined as the time from the date of surgery to death/last follow-up. Progression-free survival (PFS) is defined as the time between surgery and tumor progression, recurrence, or death.

### Statistical Analysis and Propensity Score Matching

We calculated the statistical differences of categorical variables by Chi-square test. Survival curves were constructed by Kaplan–Meier analysis with a log-rank test. Prognostic factors were identified based on univariate and multivariate Cox hazard models. Multivariate Cox proportional hazard models were constructed using a stepwise backward method. To accurately predict the OS and disease-specific survival (DSS) rates of patients with skull base chordomas, we established a prognostic nomogram system. Index of concordance (C-index) and internal validation were performed to evaluate the predictive power of the nomogram.

For reducing the substantial bias in patients selection between different groups (STR group and GTR group), we used PSM analysis, which can better evaluate the therapeutic strategies by processing relevant covariables that might affect survival independent of the extent of resection. Propensity scores were estimated using a logistic regression model based on both covariables including age, year of diagnosis, gender, race, marital status, registry, histological subtype, primary site, disease stage, radiotherapy, chemotherapy, and tumor size that might affect survival independent of the extent of resection. A 1:1 nearest neighbor matching without replacement was used with a caliper width of 0.05.

Chi-square tests and Cox regression were analyzed using the SPSS version 22.0 (IBM SPSS Statistics, Chicago, IL, United States), and *p* < 0.05 was considered statistically significant. Kaplan–Meier curves, nomogram, and PSM analysis were undertaken with the R studio based on R software (version 3.6.2). All *p*-values were two tailed.

## Results

### Overview of the Clinical Traits of Patients With Skull Base Chordoma

SEER database is a clinical follow-up database with a long time period and large sample size in multiple centers. However, skull base chordoma is a rare disease, a total of 606 patients with skull base chordoma were identified in the SEER database. There were 332 male patients (54.8%) and 274 female patients (45.2%). The age of patients ranged from 0 to 92 years, and the median age at diagnosis was 47 years. Patients aged 40–59 years comprised the largest proportion, accounting for 37.8%, and patients aged below 18 years comprised 9.9%. The median year of diagnosis was 2007, and the median follow-up time was 59 months (range 0–343 months). All selected cases were registered across 17 centers, of which the majority (453 patients, 74.8%) were treated in high-volume registries (≥30 skull base chordoma patients). Meanwhile, 41 patients did not undergo surgery (6.7%), 378 patients were treated with STR (62.4%), and 187 patients were treated with GTR (30.9%). Nearly half of the patients (49.7%) received radiation treatment. Tumor size information was available for 414 of 606 patients. The tumor size of most patients was 2–4 cm (47.6%). The 5- and 10-year OS rates of all patients were 76.8 and 61.8%, respectively. The 5- and 10-year DSS rates were 81.6 and 69.9%, respectively. The baseline characteristics of these patients are listed in Table 1.

**Table 1:** Characteristics of 606 patients with skull base chordoma tumors registered in SEER database (1973–2016).

**Patients**, ***n***	606
Median age, years (range)	47 (0–92)
Mean age, years (± SD)	45.5 (19.3)
Median follow-up time, months (range)	59 (0–343)
**Age (years)**	
<18	60 (9.9%)
18–39	169 (27.9%)
40–59	229 (37.8%)
60–79	133 (21.9%)
80+	15 (2.5%)
Year of Diagnosis	2007 median year (2006 mean)
1973–1999	119 (19.6%)
2000–2009	246 (40.6%)
2010–2016	241 (39.8%)
**Gender**	
Male	332 (54.8%)
Female	274 (45.2%)
**Race**	
White	486 (80.2%)
Black	33 (5.4%)
Others	87 (14.4%)
**Married**	
Unmarried	182 (30%)
Married	404 (66.7%)
Unknown	20 (3.3%)
**Registry**	
Low volume (<30 skull base patients)	153 (25.2%)
High volume (≥30 skull base patients)	453 (74.8%)
**Histological Subtype**	
Chordoma	541 (89.3%)
Chondroid chordoma	64 (10.6%)
Dedifferentiated chordoma	1 (0.1%)
**Primary Site**	
Bones of skull and face	440 (72.6%)
Brain	79 (13%)
Pituitary gland	34 (5.6%)
Others	53 (8.8%)
**Disease Stage**	
Localized	262 (43.2%)
Regional	233 (38.4%)
Distant	58 (9.6%)
Unknown	53 (8.8%)
**Surgery**	
No surgery performed	41 (6.7%)
STR	378 (62.4%)
GTR	187 (30.9%)
**Radiation**	
No	305 (50.3%)
Yes	301 (49.7%)
**Chemotherapy**	
No	592 (97.7%)
Yes	14 (2.3%)
**Treatment Combination**	
None	24 (3.9%)
STR only	170 (28.1%)
STR+Radiation	180 (29.7%)
GTR only	111 (18.3%)
GTR+Radiation	104 (17.2%)
Radiation only	17 (2.8%)
**Tumor Size (cm)**	
0–2	58 (9.6%)
2–4	197 (32.4%)
4–6	110 (18.2%)
>6	49 (8.1%)
Unknown	192 (31.7%)
**Overall Survival (%)**	
5-Year Overall Survival	76.8
10-Year Overall Survival	61.8
**Tumor-specific Survival (%)**	
5-Year Tumor-specific Survival	81.6
10-Year Tumor-specific Survival	69.9

*GTR, gross total resection; STR, subtotal resection*.

### Survival Analysis and Prognostic Factor Identification

**Figure 2:**
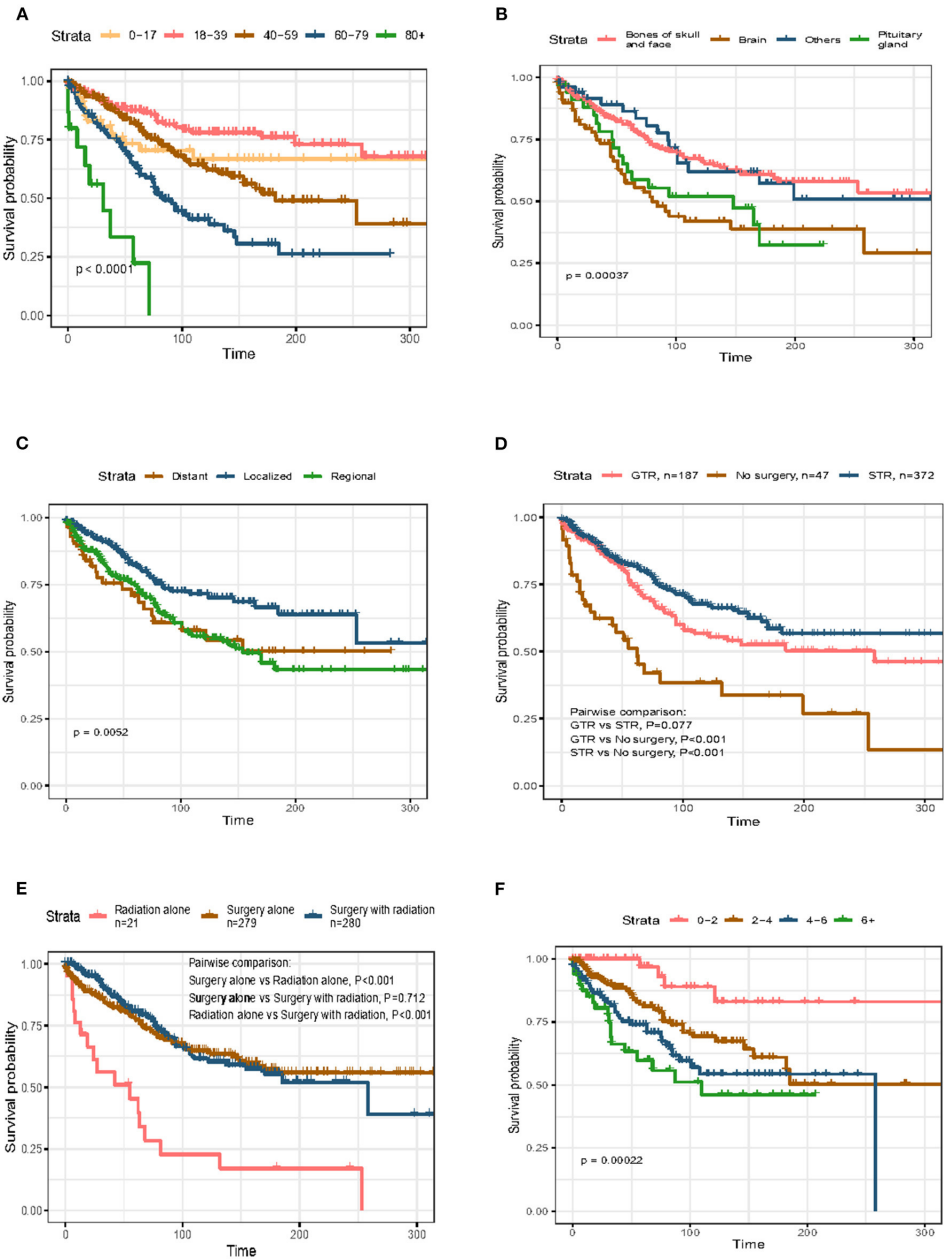
Kaplan–Meier curves (K–M curves) of overall survival (OS) in skull base chordoma patients stratified by **(A)** age, **(B)** primary site, **(C)** disease stage, **(D)** surgery, **(E)** therapy, and **(F)** tumor size.

Survival analysis based on univariate and multivariate Cox analyses was performed on all selected patients. The results showed that age at diagnosis, primary site, disease stage, tumor size, and whether or not undergo surgery were closely related to the prognosis of primary skull base chordoma (*p* < 0.05), as shown in Table 2. Multivariate analyses demonstrated that age [>80 years, hazard ratio (HR) = 8.968, 95% CI = 3.34–24.082, *p* < 0.001; 60–79 years, HR = 3.318, 95% CI = 1.514–7.269, *p* < 0.003], primary site (brain, HR = 2.198, 95% CI = 1.446–3.34, *p* < 0.001), disease stage (regional, HR = 1.594, 95% CI = 1.107–2.297, *p* = 0.012), tumor size (2–4 mm, HR = 3.457, 95% CI = 1.216–9.831, *p* = 0.02; 4–6 mm, HR = 5.148, 95% CI = 1.794–14.777, *p* = 0.002; >6 mm, HR = 5.741, 95% CI = 1.884–17.501, *P* = 0.002), and whether or not undergo surgery (STR, HR = 0.464, 95% CI = 0.253–0.852, *p* = 0.013; GTR, HR = 0.49, 95% CI = 0.267–0.901, *p* = 0.022) were independent prognostic factors for OS (Table 2). To illustrate the relationship between different risk factors and survival prognosis, the survival curves were drawn using the Kaplan–Meier analysis (Figure 2).

**Table 2:** Univariate and multivariate analysis of the prognostic factors of patients with primary skull base chordoma.

	**Univariable Cox Analysis**		**Multivariable Cox Analysis**	
**Variable**	**HR (95% CI)**	***P*** **-value**	**HR (95% CI)**	***P*** **-value**
**Age (years)**				
<18	Ref	Ref	Ref	Ref
18–39	0.571 (0.307–1.062)	0.077	0.764 (0.359–1.624)	0.484
40–59	1.072 (0.610–1.884)	0.809	1.541 (0.731–3.250)	0.256
60–79	2.118 (1.204–3.723)	0.009	3.318 (1.514–7.269)	0.003
80+	7.295 (3.237–16.440)	<0.001	8.968 (3.340–24.082)	<0.001
**Year of Diagnosis**				
1973–1999	Ref	Ref	Ref	Ref
2000–2009	0.688 (0.500–0.945)	0.021	0.977 (0.680–1.404)	0.901
2010–2016	0.360 (0.215–0.604)	<0.001	0.505 (0.285–0.894)	0.019
**Gender**				
Male	Ref	Ref	Ref	Ref
Female	0.931 (0.694–1.249)	0.635	0.939 (0.686–1.286)	0.695
**Race**				
White	Ref	Ref	Ref	Ref
Black	0.842 (0.429–1.651)	0.617	1.408 (0.687–2.887)	0.35
Others	1.056 (0.691–1.614)	0.8	1.172 (0.750–1.830)	0.486
**Married**				
Unmarried	Ref	Ref	Ref	Ref
Married	1.375 (0.972–1.946)	0.072	0.943 (0.606–1.467)	0.793
Unknown	1.924 (0.903–4.100)	0.09	1.592 (0.703–3.604)	0.264
**Registry**				
Small	Ref	Ref	Ref	Ref
Large	0.853 (0.618–1.177)	0.333	0.854 (0.605–1.205)	0.368
**Histological Subtype**				
Chordoma	Ref	Ref	Ref	Ref
Chondroid chordoma	0.515 (0.280–0.948)	0.033	0.488 (0.259–0.919)	0.026
Dedifferentiated chordoma	–	–	–	–
**Primary Site**				
Bones of skull and face	Ref	Ref	Ref	Ref
Brain	2.037 (1.408–2.947)	<0.001	2.198 (1.446–3.340)	<0.001
Pituitary gland	1.778 (1.079–2.928)	0.024	1.108 (0.646–1.903)	0.709
Others	0.990 (0.585–1.675)	0.97	0.867 (0.495–1.519)	0.617
**Disease Stage**				
Localized	Ref	Ref	Ref	Ref
Regional	1.706 (1.210–2.407)	0.002	1.594 (1.107–2.297)	0.012
Distant	1.735 (1.055–2.854)	0.03	1.282 (0.750–2.193)	0.364
Unknown	1.724 (1.054–2.820)	0.03	1.078 (0.622–1.869)	0.788
**Extent of Surgical Resection**				
No surgery performed	Ref	Ref	Ref	Ref
STR	0.333 (0.212–0.521)	<0.001	0.464 (0.253–0.852)	0.013
GTR	0.429 (0.268–0.689)	<0.001	0.49 (0.267–0.901)	0.022
**Radiation**				
No	Ref	Ref	Ref	Ref
Yes	1.041 (0.777–1.395)	0.787	0.984 (0.430–2.250)	0.97
**Chemotherapy**				
No	Ref	Ref	Ref	Ref
Yes	1.968 (0.871–4.445)	0.104	2.628 (0.987–6.998)	0.053
**Tumor Size (cm)**				
0–2	Ref	Ref	Ref	Ref
2–4	3.368 (1.206–9.405)	0.02	3.457 (1.216–9.831)	0.02
4–6	5.142 (1.827–14.469)	0.002	5.148 (1.794–14.777)	0.002
>6	6.983 (2.375–20.533)	<0.001	5.741 (1.884–17.501)	0.002
Unknown	4.857 (1.780–13.254)	0.002	4.338 (1.546–12.171)	0.005

*GTR, gross total resection; STR, subtotal resection*.

Survival analysis showed no significant difference in OS between patients receiving GTR and those receiving STR (*p* = 0.077; Figure 2D). As the distributions of the year of diagnosis and patients' race were significantly different between the GTR and STR groups (*p* = 0.037 and *p* = 0.020, respectively; Table 3), PSM was conducted to minimize the selection bias (Table 4). Subsequently, 168 pairs of patients were generated and both covariables entered in PSM were balanced (all *p* > 0.05). After PSM, the OS difference between patients with GTR and those with STR was still insignificant (*p* = 0.157). The 5- and 10-year OS rates of patients with GTR were 74.5 and 54.7%, and the 5- and 10-year OS rates of patients with STR were 80.6 and 65.3%, respectively.

**Table 3:** Comparison of patients with skull base chordomas receiving GTR or STR.

**Postoperative Radiation**	**GTR**	**STR**	***P*** **-value**
Total number of patients	187	378	
**Age (years)**			0.362
<18	17 (9.1%)	41 (10.9%)	
18–39	51 (27.3%)	109 (28.8%)	
40–59	70 (37.4%)	147 (38.9%)	
60–79	42 (22.5%)	76 (20.1%)	
80+	7 (3.7%)	5 (1.3%)	
**Year of Diagnosis**			0.037
1973–1999	47 (28.1%)	61 (16.1%)	
2000–2009	72 (38.5%)	159 (42.1%)	
2010–2016	68 (36.4%)	158 (41.8%)	
**Gender**			0.915
Male	103 (55.1%)	210 (55.6%)	
Female	84 (44.9%)	168 (44.4%)	
**Race**			0.020
White	151 (80.7%)	300 (79.3%)	
Black	16 (8.6%)	15 (4%)	
Others	20 (10.7%)	63 (16.7%)	
**Marital Status**			0.833
Unmarried	56 (30%)	117 (30.9%)	
Married	125 (66.8%)	252 (66.7%)	
Unknown	6 (3.2%)	9 (2.4%)	
**Registry**			0.578
Small	43 (23%)	95 (25.1%)	
Large	144 (77%)	283 (74.9%)	
**Histological Subtype**			0.780
Chordoma	167 (89.3%)	337 (89.1%)	
Chondroid chordoma	20 (10.7%)	40 (10.6%)	
Dedifferentiated chordoma	0 (0%)	1 (0.3%)	
**Primary Site**			0.180
Bones of skull and face	126 (67.4%)	287 (75.9%)	
Brain	29 (15.5%)	42 (11.1%)	
Pituitary gland	14 (7.5%)	19 (5%)	
Others	18 (9.6%)	30 (8%)	
**Disease Stage**			0.052
Localized	74 (39.6%)	182 (48.1%)	
Regional	73 (39%)	145 (38.4%)	
Distant	24 (12.8%)	26 (6.9%)	
Unknown	16 (8.6%)	25 (6.6%)	
**Radiotherapy**			0.721
No	95 (50.8%)	186 (49.2%)	
Yes	92 (49.2%)	192 (50.8%)	
**Chemotherapy**			0.986
No	183 (97.9%)	370 (97.9%)	
Yes	4 (2.1%)	8 (2.1%)	
**Tumor Size (cm)**			0.327
0–2	19 (10.1%)	108 (28.6%)	
2–4	58 (31.0%)	39 (10.3%)	
4–6	31 (16.5%)	135 (35.7%)	
>6	19 (10.1%)	73 (19.3%)	
Unknown	60 (32.0%)	23 (6.1%)	
**Overall Survival (%)**			0.104
5-Year Overall Survival	74.1	81.2	
10-Year Overall Survival	56.6	67.0	
**Tumor-specific Survival (%)**			0.316
5-Year Tumor-specific Survival	81.7	84.3	
10-Year Tumor-specific Survival	65.6	74.5	

*GTR, gross total resection; STR, subtotal resection*.

**Table 4:** Comparison of patients with skull base chordomas receiving GTR or STR after propensity score matching.

**Postoperative Radiation**	**GTR**	**STR**	***P*** **-value**
Total number of patients	168	168	
**Age (years)**			0.950
<18	17 (10.1%)	18 (10.7%)	
18–39	48 (28.6%)	45 (26.8%)	
40–59	61 (36.3%)	63 (37.5%)	
60–79	37 (22%)	39 (23.2%)	
80+	5 (3%)	3 (1.8%)	
**Year of Diagnosis**			0.856
1973–1999	35 (20.8%)	33 (19.6%)	
2000–2009	66 (39.3%)	71 (42.3%)	
2010–2016	67 (39.9%)	64 (38.1%)	
**Gender**			1
Male	94 (56%)	94 (56%)	
Female	74 (44%)	74 (44%)	
**Race**			0.150
White	134 (79.8%)	137 (81.5%)	
Black	14 (8.3%)	6 (3.6%)	
Others	20 (11.9%)	25 (14.9%)	
**Marital Status**			0.775
Unmarried	52 (30.9%)	50 (29.8%)	
Married	110 (65.5%)	114 (67.8%)	
Unknown	6 (3.6%)	4 (2.4%)	
**Registry**			0.694
Small	39 (23.2%)	36 (21.4%)	
Large	129 (76.8%)	132 (78.6%)	
**Histological Subtype**			0.736
Chordoma	149 (88.7%)	147 (87.5%)	
Chondroid chordoma	19 (11.3%)	21 (12.5%)	
Dedifferentiated chordoma	0 (0%)	0 (0%)	
**Primary Site**			0.889
Bones of skull and face	122 (72.6%)	127 (75.6%)	
Brain	20 (11.9%)	18 (10.7%)	
Pituitary gland	12 (7.2%)	9 (5.4%)	
Others	14 (8.3%)	14 (8.3%)	
**Disease Stage**			0.326
Localized	74 (44%)	75 (44.6%)	
Regional	70 (41.7%)	67 (39.8%)	
Distant	18 (10.7%)	13 (7.8%)	
Unknown	6 (3.6%)	13 (7.8%)	
**Radiotherapy**			0.445
No	82 (49%)	89 (53%)	
Yes	86 (51%)	79 (47%)	
**Chemotherapy**			1
No	164 (97.6%)	164 (97.6%)	
Yes	4 (2.4%)	4 (2.4%)	
**Tumor Size (cm)**			0.452
0–2	53 (31.6%)	59 (35.1%)	
2–4	18 (10.7%)	16 (9.5%)	
4–6	54 (32.1%)	51 (30.4%)	
>6	25 (14.9%)	32 (19%)	
Unknown	18 (10.7%)	10 (6%)	
**Overall Survival (%)**			0.157
5-Year Overall Survival	74.5	80.6	
10-Year Overall Survival	54.7	65.3	
**Tumor-specific Survival (%)**			0.475
5-Year Tumor-specific Survival	82.4	83.2	
10-Year Tumor-specific Survival	62.9	72.8	

*GTR, gross total resection; STR, subtotal resection*.

### Estimation of Identified Prognostic Factor Efficiency by Nomograms

**Figure 3:**
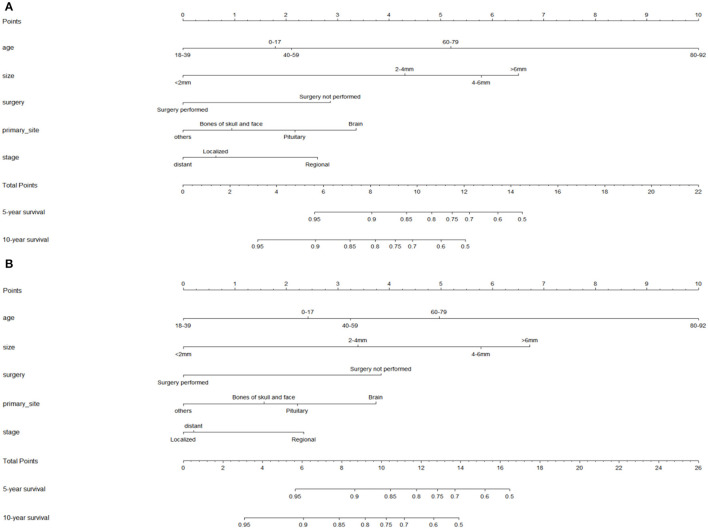
Nomograms for predicting the 5- and 10-year overall survival **(A)** and disease-specific survival (DSS) **(B)** of skull base chordoma patients. OS index of concordance (C-index): 0.764, DSS C-index: 0.755.

**Figure 4:**
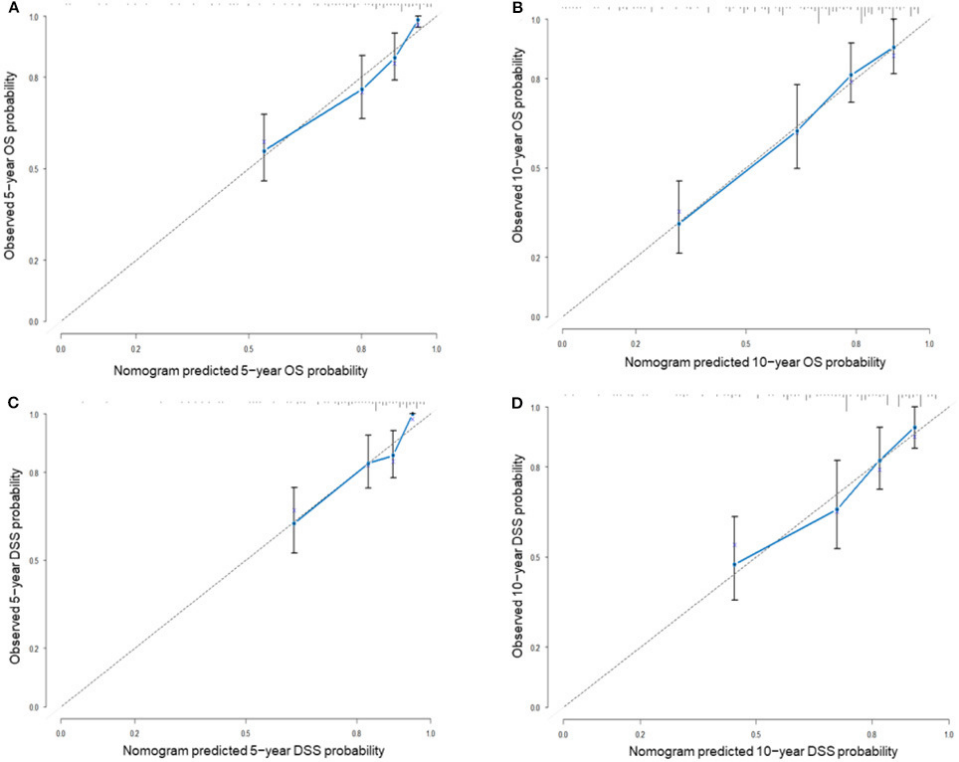
The calibration plots for internal validation of **(A)** actual 5-year OS and **(B)** 10-year OS; **(C)** actual 5-year DSS and **(D)** actual 10-year DSS. The 45-degree dashed line represents an ideal match between the actual observed survival (Y-axis) and nomogram-predicted survival (X-axis). The perpendicular line represents 95% confidence intervals. Closer distances from the points to the dashed line mean a higher prediction accuracy.

Nomograms predicting 5- and 10-year OS and DSS were constructed based on statistically significant factors identified by multivariate Cox analysis (Figure 3). Age, primary site, tumor size, surgical treatment, and disease stage were included as prognostic predictors in the nomograms. By adding the scores for each selected variable, the survival probability of each individual was easily calculated. The C-indexes were high in both internal validations (OS: 0.764, 95% CI, 0.733–0.795; DSS: 0.755, 95% CI, 0.724–0.785). The calibration plots for the probability of OS and DSS at 5- and 10-years showed an optimal agreement between the prediction by nomogram and the actual observation (Figure 4).

### Validating the Impact of Surgical Resection and Adjuvant Radiotherapy in an Independent Data Set

**Figure 5:**
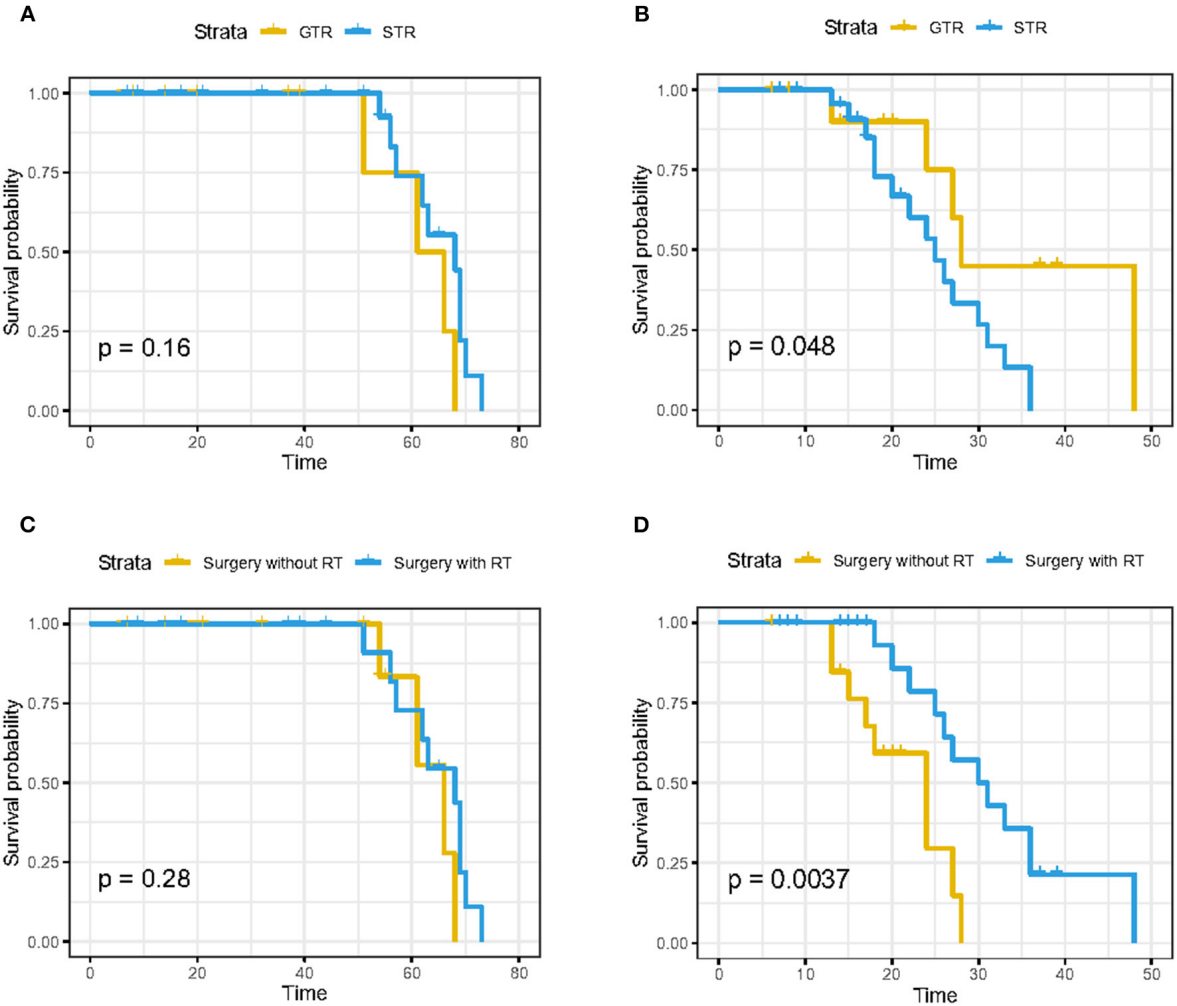
Kaplan–Meier curves of OS in patients from Xiangya Hospital stratified by **(A)** gross total resection (GTR) and subtotal resection (STR), **(C)** surgery with radiotherapy and surgery without radiotherapy; K–M curves of progression-free survival in patients from Xiangya Hospital stratified by **(B)** GTR and STR and **(D)** surgery with radiotherapy and surgery without radiotherapy.

Another 37 skull base chordoma patients' clinical data from the Xiangya Hospital were used as the validation data set. the baseline characteristics of these patients are demonstrated in Table 5. According to the extent of surgical resection, 13 cases (35.1%) underwent GTR and 24 cases (64.9%) underwent STR. In addition, 22 patients (59.5%) received stereotactic radiotherapy, while 15 patients (40.5%) did not receive radiotherapy. Results of survival analysis showed no statistically significant difference in OS between GTR and STR groups (*p* = 0.16; Figure 5A). For PFS, the GTR group was superior to the STR group and the difference was statistically significant (*p* = 0.048; Figure 5B). There was no statistically significant difference in OS between patients who received stereotactic radiotherapy after surgery and those who did not receive radiotherapy (*p* = 0.28; Figure 5C), while the PFS of patients who received radiotherapy was significantly better than that of patients who did not receive radiotherapy (*p* = 0.0037; Figure 5D). These results indicate that GTR does not appear to improve OS, but it is associated with improvements in PFS. At the same time, although postoperative radiotherapy does not improve OS, it can prolong the time of PFS and reduce the recurrence rate.

**Table 5:** Characteristics of 37 patients with skull base chordoma treated in Xiangya Hospital (2010–2020).

**Age (years)**	
Mean (SD)	46.8 (12.4)
Median [Min, Max]	48.0 [13.0, 68.0]
**Gender**	
Female	15 (40.5%)
Male	22 (59.5%)
**Histological subtype**	
Chordoma	37 (100%)
**Primary site**	
Bones of skull	18 (48.6%)
Brain	7 (18.9%)
Pituitary gland	12 (32.4%)
**Disease stage**	
Distant	7 (18.9%)
Local	5 (13.5%)
Region	25 (67.6%)
**Surgery**	
GTR	13 (35.1%)
STR	24 (64.9%)
**Radiation**	
None	15 (40.5%)
Radiation after surgery	22 (59.5%)
**Tumor size (cm)**	
Mean (SD)	4.11 (1.21)
Median [Min, Max]	4.00 [2.30, 8.00]

*GTR, gross total resection; STR, subtotal resection*.

## Discussion

By analyzing 606 cases of skull base chordomas from the SEER database and validated by an independent data set from the Xiangya Hospital, this study found that the patient's age, tumor size, surgery, primary location, and tumor stage were closely related to the patient's prognosis (5, 6). Previous studies reported a different age of onset of skull base chordomas. Crockard reported that the average age was 58.1-years, while Al-Mefty reported an average age of 38.4-years, and Wu reported an average age of 35.3-years (16–18). Anyhow, age is an important factor affecting the patients' prognosis, and the prognosis of elderly patients is worse than that of young patients, for elderly patients are more likely to suffer recurrence and metastasis (16, 19). Moreover, elderly patients have poor tolerance to surgery and are more likely to have serious postoperative complications that affect prognosis. In addition, due to the slow growth of tumors and the larger space for intracranial compensation caused by brain atrophy in elderly patients, neurological symptoms tend to appear lately and atypically. When the symptom appears, the tumor is usually very large and has already adhered to the surrounding brain stem or important vessels and nerves. Such patients have a higher risk of surgical resection and tend to suffer serious complications (20).

Compared with sacrococcygeal chordoma, the prognosis of skull base chordoma is more sensitive to tumor size (21, 22). Tumor size is mainly related to tumor stage and classification. For example, the classification of Al-Mefty is defined as Type I: Small tumor with mild symptoms or no symptom and limited to a single anatomical space at the skull base (such as sphenoid sinus, cavernous sinus, inferior slope, or occipital condyle). Type II: The tumor is large and invades two or more anatomical spaces of the skull base, which can be removed through a skull base approach. Type III: Extensive infiltration of skull base multiple anatomical spaces, need to be combined with two or more skull base approaches for total resection of the tumor (17). The larger the tumor, the more extensive is the scope of invasion. Thus, surgical resection becomes more difficult, with more complications, and the prognosis will be worse. Meanwhile, according to the staging method of Wu, Stage I indicates that the tumor is primary and confined to a certain site. It is completely epidural, with no intracranial invasion and only mild neurological dysfunction. Stage II: the primary tumor is mainly epidural, but local dural invasion causes intracranial structure compression, with mild-to-moderate neurological dysfunction. Stage III: primary tumor growth is widespread, mostly invades subdural, compressing or adhering to the brain stem, with medium-to-severe neurological dysfunction. Stage IV: The tumor is metastatic, located either in the epidural or subdural, with or without neurological dysfunction (18). Although the staging method does not directly mention the relationship between tumor size and tumor staging, larger tumors are more likely to break through the dura, adhere to the brainstem and cause more severe neurological dysfunction (7). Furthermore, the closer the primary site of the tumor is to the brainstem or the vascular and nerve of the skull base, the greater the risk of treatment, with more complications and worse prognosis.

The prognosis of patients receiving surgical treatment was significantly better than that of patients without surgical treatment. However, analysis based on the SEER database found no statistically significant difference in OS between patients undergoing GTR and those undergoing STR after PSM. The results by validating data from the Xiangya Hospital also indicated that there was no statistical difference in OS between GTR and STR patients, but PFS could be improved. This suggests that GTR is not mandatory for skull base chordoma, and it is difficult to achieve safe resection, especially for tumors invading the subdural and adhering to the brainstem or important nerves and vessels. Forced GTR may cause serious postoperative complications, and lead to a worse prognosis (20, 23). At the same time, according to the therapeutic guidelines of skull base chordoma, if the total resection of the tumor is difficult, the purpose of surgery is to relieve the compression of the tumor on the brain stem and optic pathway, reduce the tumor volume, and lay the foundation for improving the efficacy of subsequent radiotherapy (24). Therefore, it should be advocated to remove as many tumors as possible under the premise of safety. At present, endoscopy is increasingly applied to the surgical treatment of skull base chordoma. Endoscopy can show locations that cannot be observed by microscope, providing a better vision for safe and thorough resection of tumors (6, 25–27). Staged resection can be performed for some tumors with a wide range of growth. Recently, some neurosurgeons have tried simultaneous combined surgery, combining transnasal and transcranial approaches with endoscopy, which can improve the safety and timeliness of surgery (28, 29). Moreover, intraoperative vascular ultrasound, neuronavigation, and neuroelectrophysiological monitoring can improve the accuracy and safety of surgery (30, 31).

The SEER database analysis showed that postoperative radiotherapy for prognosis improvement was not statistically significant, consistent with previous reports that chordoma was not sensitive to ordinary radiotherapy, mainly because it could not reach the effective radiation dose (32). However, according to the current treatment guidelines and expert consensus, adjuvant radiotherapy should be performed in patients with skull base chordoma after surgery, especially for patients who did not achieve total resection of the tumor. Our study based on the clinical data from the Xiangya Hospital found that although postoperative radiotherapy could not improve the OS rate, it could significantly prolong the PFS and had a positive effect on reducing the recurrence rate. With the development and popularization of new radiotherapy techniques, especially stereotactic radiotherapy and three-dimensional conformal radiotherapy, radiotherapy is more accurate for lesions, which increases the dose of tumor irradiation and reduces the damage to the surrounding normal brain tissue and cranial nerves (33, 34). Furthermore, proton beam radiotherapy or proton plus photon radiotherapy also has a good therapeutic effect on chordoma (35–38). Numerous studies have suggested that neo-adjuvant radiotherapy, as adjuvant therapy, can control the local recurrence of tumors to a certain extent and improve the prognosis of patients when the chordoma is subtotally resected or the resection margin is not clean (21, 39).

Given the insensitivity of chordoma to conventional chemotherapy, molecular targeted therapy is particularly important. Some recent studies have found that molecular targeting technology has a good prospect in the treatment of chordoma. At present, the research on the molecular mechanism of chordoma mainly includes receptor tyrosine kinase and its downstream signaling pathways, Src/Stat3 signaling pathway and PI3K/AKT/mTOR pathway (40–44). Many clinical studies on tyrosine kinase inhibitors, such as imatinib and erlotinib, suggested that tyrosine kinase inhibitors may be the breakthrough in the treatment of chordoma (45, 46).

This study had some limitations. Since this was a retrospective study of the SEER database, some clinical data were not detailed enough, such as the description of the primary site of the tumor was not accurate enough, and the SEER database lacks postoperative images which may concern patients' privacy, it is impossible to confirm whether the resection range meets the clinical guidelines. Although our data from the Xiangya Hospital included relatively detailed image data, the sample number was limited due to the rare incidence of this disease. Hence, multi-center prospective cohort studies with large sample sizes are needed in the future.

## Conclusions

The main factors affecting the prognosis of chordoma of the skull base include the age of the patients, tumor size, surgical treatment, primary site, and tumor stage (5, 6). The main treatment for skull base chordoma remains surgical resection. Survival analysis showed no significant difference between GTR and STR for OS, GTR can significantly improve PFS, but it is difficult to achieve safely total resection because of its close relationship with brain stem and important cranial nerves and vessels. Forced GTR may lead to serious postoperative complications and affect the prognosis of patients. Therefore, resection of the tumor as much as possible while ensuring safety, combined with postoperative neo-adjuvant radiotherapy may be an ideal treatment for skull base chordoma. In addition, molecular targeted therapy is a very promising and important treatment for chordoma in the future.

## Data Availability Statement

The raw data supporting the conclusions of this article will be made available by the authors, without undue reservation.

## Ethics Statement

The studies involving human participants were reviewed and approved by Ethics Committee of Xiangya Hospital of Central South University. Written informed consent to participate in this study was provided by the participants' legal guardian/next of kin.

## Author Contributions

CT conceived and designed the experiments, collected the data, prepared tables, authored and reviewed drafts of the article, and approved the final draft. QY and ZX analyzed the data, prepared figures and tables, and approved the final draft. NY prepared figures and tables and approved the final draft. XL conceived and designed the experiments, authored and reviewed drafts of the article, and approved the final draft. All authors contributed to the article and approved the submitted version.

## Funding

This work was supported by the National Natural Science Foundation of China (for XL, Grant Nos. 81770781 and 81472594) and Natural Science Foundation of Hunan Province, China (Grant No. 2019JJ50978).

## Conflict of Interest

The authors declare that the research was conducted in the absence of any commercial or financial relationships that could be construed as a potential conflict of interest.

## Publisher's Note

All claims expressed in this article are solely those of the authors and do not necessarily represent those of their affiliated organizations, or those of the publisher, the editors and the reviewers. Any product that may be evaluated in this article, or claim that may be made by its manufacturer, is not guaranteed or endorsed by the publisher.
